# Molecular Mechanisms of SGLT2 Inhibitor on Cardiorenal Protection

**DOI:** 10.3390/ijms21217833

**Published:** 2020-10-22

**Authors:** Yi-Chou Hou, Cai-Mei Zheng, Tzung-Hai Yen, Kuo-Cheng Lu

**Affiliations:** 1 Division of Nephrology, Department of Medicine, Cardinal-Tien Hospital, School of Medicine, Fu Jen Catholic University, New Taipei City 234, Taiwan; d118106004@tmu.edu.tw; 2 Graduate Institute of Clinical Medicine, School of Medicine, College of Medicine, Taipei Medical University, Taipei 110, Taiwan; 3 Division of Nephrology, Department of Internal Medicine, Taipei Medical University Shuang Ho Hospital, New Taipei City 235, Taiwan; 11044@s.tmu.edu.tw; 4 Division of Nephrology, Department of Internal Medicine, School of Medicine, College of Medicine, Taipei Medical University, Taipei 110, Taiwan; 5 Taipei Medical University-Research Center of Urology and Kidney, Taipei Medical University, Taipei 110, Taiwan; 6 Division of Nephrology, Department of Internal Medicine, Chang Gung Memorial Hospital, Taipei 105, Taiwan; m19570@adm.cgmh.org.tw; 7 Department of Medicine, College of Medicine, Chang Gung University, Taoyuan 333, Taiwan; 8 Division of Nephrology, Department of Medicine, Taipei Tzu Chi Hospital, Buddhist Tzu Chi Medical Foundation, New Taipei City 231, Taiwan

**Keywords:** cardiovascular disease, chronic kidney disease, SGLT2, diabetes mellitus

## Abstract

The development of sodium-glucose transporter 2 inhibitor (SGLT2i) broadens the therapeutic strategies in treating diabetes mellitus. By inhibiting sodium and glucose reabsorption from the proximal tubules, the improvement in insulin resistance and natriuresis improved the cardiovascular mortality in diabetes mellitus (DM) patients. It has been known that SGLT2i also provided renoprotection by lowering the intraglomerular hypertension by modulating the pre- and post- glomerular vascular tone. The application of SGLT2i also provided metabolic and hemodynamic benefits in molecular aspects. The recent DAPA-CKD trial and EMPEROR-Reduced trial provided clinical evidence of renal and cardiac protection, even in non-DM patients. Therefore, the aim of the review is to clarify the hemodynamic and metabolic modulation of SGLT2i from the molecular mechanism.

## 1. Introduction

Sodium–glucose cotransporter (SGLT) 2 inhibitors (SGLT2i) are essential in the therapeutic management of diabetic nephropathy. Their role in lowering the mortality associated with congestive heart failure and in alleviating the glomerular filtration rate (GFR) decline has been proven by multiple landmark clinical trials. They improve mortality by reducing the sodium and glucose load on the body. In vivo studies have demonstrated their pleiotropic effects in addition to the restoration of sodium/fluid homeostasis, including calcium/phosphate homeostasis, magnesium levels, glomerular tubular feedback, and energy metabolism.

This study reviews the pleiotropic effect of SGLT2i, particularly in the management of cardiorenal syndrome based on molecular mechanisms.

## 2. Mechanism of Glomerular Hyperfiltration in Diabetes

The kidney is a critical organ that manages glucose homeostasis. As renal arteries carry glucose into the kidney, the renal medulla uptakes the circulating glucose and uses it as an energy source. At the same time, the renal cortex produces glucose through gluconeogenesis that can be utilized by the renal medulla [1]. SGLT2s are distributed mainly in renal proximal tubular cells. The filtered sodium and glucose from the glomerulus are mainly reabsorbed by SGLT2 and GLUT2 within the S1–2 segment of the proximal convoluted tubules (PTs). SGLT2 and GLUT2 govern 90% of the reabsorption of glucose within the S1 segment. Kidneys play a role in glucose homeostasis in the body by ensuring that glucose is not lost in the urine. Epithelial cells of the S3 segment express SGLT1 on the apical membrane and GLUT2 on the basolateral membrane. In both S1/S2 and S3 segments, glucose reabsorption occurs, first via glucose transport across the apical membrane by SGLTs and then by passive glucose exit towards the plasma via GLUT2. The sodium gradient across the apical membrane is maintained by the basolateral Na^+^/K^+^-adenosine triphosphatase, which pumps out Na^+^ and pumps in K^+^ resulting in maintaining low intracellular Na^+^ concentration, thereby facilitating glucose reabsorption through the luminal membrane SGLTs [2].

In diabetes mellitus (DM), both hyperinsulinemia and hyperglycemia alter the phenotype of PTs by modulating SGLT2 [3]. In animal models of type 1 DM (T1DM) and type 2 DM (T2DM), SGLT2 protein expression increased by up to 40–80%. Both SGLT2 and SGLT1 exist in PTs. SGLT1 mediates 3% of total glucose reabsorption in the S3 segment. SGLT2 inhibition causes a compensatory increase in SGLT1 expression, thus increasing glucose reabsorption from PTs [4]. Thus, the risk of hypoglycemia is low. The hyperglycemic state stimulates glucose uptake by PT cells through GLUT-2, which is located at the basolateral side. The increased intracellular glucose concentration stimulates SGLT2 expression by lowering Sirt-1 expression, which increases glucose uptake from PTs. Hyperinsulinemia also directly stimulates SGLT2 expression [5].

Diabetic nephropathy involves functional and structural alterations of the glomeruli and renal tubules in DM. The initial phase of diabetic nephropathy is characterized by glomerular hyperfiltration [6]. As type 1 diabetes mellitus progresses, the kidneys enlarge; this phenomenon is most likely caused by growth factors, although obesity may also be an independent contributor [7]. Some studies have suggested that compensatory hypertrophy results from hyperfiltration; however, animal studies have demonstrated that hypertrophy develops before hyperfiltration. In the setting of glomerular hypertrophy, efferent arterioles constrict and afferent arterioles dilate to maintain a single-nephron GFR. Simultaneously, Na^+^/H^+^ exchanger isoform 3 (NHE3) expression in PTs increases, leading to a decline in afferent arteriolar resistance and an increase in the single-nephron GFR by inhibiting tubuloglomeular feedback (TGF). An excessive increase in the GFR downregulates the tubular glomerular feedback [8]. In the event of volume depletion or decreased renal perfusion, decreased GFR lowers the urinary flow into the macula densa. Consequently, the juxtaglomerular apparatus reduces adenosine secretion and enhances afferent arteriolar vasodilation to maintain the GFR. During hyperfiltration, TGF is suppressed to reduce urinary flow to the macula densa. In addition to afferent arteriolar vasoconstriction, hypertrophy within PT cells is associated with SGLT2 overexpression because enhanced glucose reabsorption reduces urine flow.

Normally, the proximal tubule isosmotically reabsorbs 60–70% of filtered NaCl and water and 70–90% of filtered bicarbonate [9]. A significant portion (approximately 30%) of proximal tubular Na^+^ reabsorption occurs through direct or indirect action of apical NHE3 [10]. NHE3 contributes to the primary tubular hyper-reabsorption and is therefore responsible for diabetes-associated hyperfiltration and a rise in blood pressure [11]. In diabetic patients, the increase in filtered glucose enhances SGLT2 activity, which worsens glycemic control and promotes Na^+^ loading with subsequent impaired blood pressure control [12]. Enhanced proximal tubular Na^+^ reabsorption results in decreased Na^+^ availability at distal tubules and reduces macula densa adenosine signaling signaling to afferent arterioles. The signal reduction leads to afferent arteriole vasodilation, increases intraglomerular pressure, and causes hyperfiltration. Further, studies in human proximal tubular cells have indicated a link between NHE3-mediated cellular Na^+^ uptake and tubular growth under pathophysiologic concentration of albumin [13]. NHE3 is closely regulated by glucose metabolism and SGLT transporters, which makes it sensitive to SGLT2 inhibitors [14]. Vallon et al. revealed the effects of renal tubular NHE3 knockdown in type 1 diabetic Akita mice. HE3-KO mice were also associated with lower SGLT2 and higher SGLT1 expression [15]. In diabetic mice, a chronic SGLT2 inhibitor enhanced phosphorylation of NHE3, resulting in less NHE3-mediated tubular Na^+^ reabsorption [16]. In Otsuka Long-Evans Tokushima Fatty diabetic rats, SGLT2 inhibitor empagliflozin decreased the tubular expression of NHE3 and the epithelial Na^+^ channels [17]. Thus, part of the natriuretic response to an SGLT2 inhibitor was mediated by suppressing NHE3 [18]. To summarize, SGLT2 inhibitors not only downregulates SGLT2 but also decreases sodium uptake by NHE3, thus lowering both the reabsorption of sodium and glucose, with consequent improvement in fluid retention and hyperglycemia status and reestablishment of the tubular glomerular feedback.

The vasoconstriction of efferent arterioles is mostly activated by angiotensin II. Angiotensin II also induces extracellular matrix (ECM) synthesis and mesangial cell growth, and this aberrant ECM growth increases the synthesis of fibronectin or laminin, leading to ECM fibrosis. However, nephrin or proteoglycan expression is suppressed, leading to glomerular capillary defects.

## 3. Hemodynamic Factors Affected by SGLT2i

A clinical trial involving patients with T2DM having cardiovascular disease revealed that SGLT2i attenuates cardiovascular events, reduces all-cause mortality [19], and potentially reduces hospitalization rates for heart failure. Another study revealed that SGLT2i in patients provided renoprotection in T2DM patients with high cardiovascular risk [20]. Unlike traditional glucose-lowering agents, SGLT2i provides cardiac and renal protective effects in addition to improvements in blood glucose, lipid levels, and blood pressure control. Thus, hemodynamic changes associated with SGLT2i may play a role in alleviating further renal damage.

### 3.1. Improved Glomerular Hyperfiltration and Renal Hemodynamics

#### 3.1.1. Renal Hemodynamic Effects of SGLT2i in Patients with T1DM

**Figure 1:**
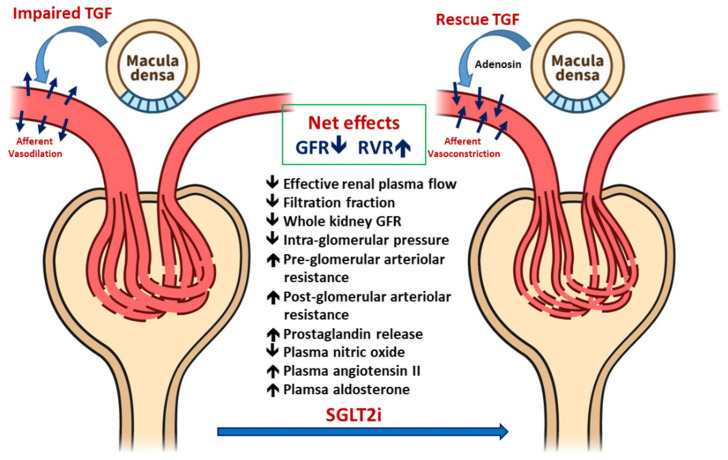
Hemodynamic responses to a sodium-glucose transporter 2 inhibitor (SGLT2i) in patients with early stage type 1 diabetes mellitus (T1DM): in the early stage of T1DM, increased proximal tubular reabsorption will decrease the distal delivery resulting in impaired tubular glomerular feedback (TGF) which presents with dilated glomerular afferent arteriolar and increased intraglomerular pressure. SGLT2i treatment will increase solute and fluid delivery to macula densa, which will enhance the secretion of adenosine, thereby increasing the afferent arteriolar resistance resulting in reducing the GFR and intraglomerular pressure. The net effects will be slightly decreased in GFR and will increase renal vascular resistance.

Figure 1 demonstrated the renal hemodynamic change influenced by SGLT2i in T1DM patients. TGF signaling maintains the GFR by modulating the preglomerular vascular tone [21]. Preglomerular vasodilatation increases glomerular filtration, which increases the distal tubular sodium delivery to the macula densa within the juxtaglomerular apparatus, which then adjusts the GFR [22]. In chronic hyperglycemia, this feedback mechanism is impaired because of the disturbed myogenic-mediated response in afferent arterioles [23] and increased proximal SGLT2 expression [24]. SGLT2 overexpression increases proximal reabsorption of sodium and glucose and therefore reduces sodium exposure to the macula densa despite an increase in the GFR due to hyperfiltration [25]. This impairment of TGF signaling likely leads to an inadequate attenuated arteriole tone, resulting in increased renal perfusion. SGLT2i treatment attenuates PT glucose and sodium reabsorption, thus maintaining sodium delivery to the macula densa [26]. Furthermore, TGF is restored by an appropriate increase in the afferent arteriolar tone, which in turn reduces renal plasma flow and hyperfiltration. In T1DM, excessive glucose delivery induces a whole-kidney GFR increase, which is reversed by insulin treatment [27].

In a clinical trial evaluating the effects of 8-week SGLT2i treatment on renal hyperfiltration in young adults with T1DM, SGLT2i attenuated hyperfiltration (GFR ≥ 135 mL/min/1.73 m^2^) by lowering the GFR (−33 mL/min/1.73 m^2^) if participants had a clamped euglycemia state (blood sugar, 4–6 mmol/L). The plasma and effective renal plasma NO decreased along with an increase in renal vascular resistance. By contrast, plasma NO and GFR were similar in normal healthy participants after treatment with SGLT2i. The results indicated that short-term SGLT2i treatment reduces renal hyperfiltration in patients with T1DM, likely by affecting TGF signaling [21].

#### 3.1.2. Renal Hemodynamic Effect of SGLT2i in Patients with T2DM 

**Figure 2:**
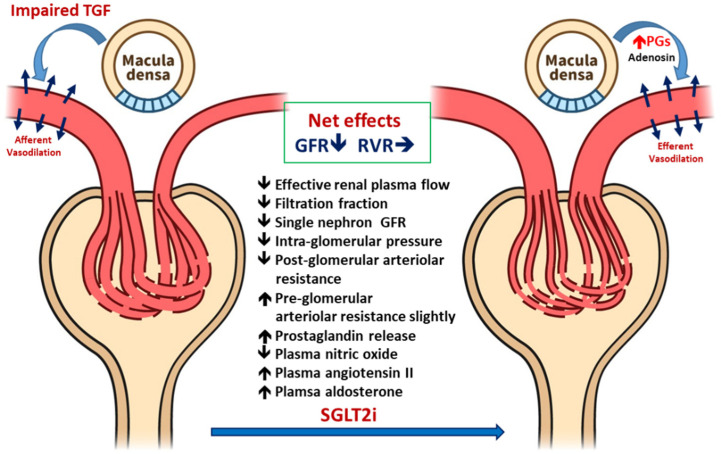
Hemodynamic responses to SGLT2i in patients with early stage type 2 diabetes mellitus (T2DM): in the early stage of T2DM, increased proximal tubular reabsorption will decrease the distal delivery resulting in impaired tubular glomerular feedback (TGF), which present with dilated glomerular afferent arteriolar and increased intraglomerular pressure. SGLT2i treatment will increase solute and fluid delivery to macula densa, which will enhance the secretion of prostaglandins resulting in dilated glomerular efferent arterioles. It also increased the adenosine level trivially, which may slightly increase afferent arteriolar resistance. Both factors reduce the intraglomerular pressure. The net effects will be slightly decreased in GFR but will adequately maintain renal vascular resistance.

Figure 2 illustrated the renal hemodynamic change influenced by SGLT2i in T2DM patients. In T2DM, the hemodynamic mechanism of hyperfiltration is different from T1DM-mediated hyperfiltration. In diabetic nephropathy, GFR might be normal or even decreased with increased single-nephron GFR in the hyperfiltration state [5]. van Bommel et al. compared the effects of dapagliflozin with gliclazide in patients with T2DM to study the renal hemodynamic effects of SGLT2i [28]. Using the Gomez equations to estimate glomerular hemodynamic data in T2DM with hyperfiltration, they found that dapagliflozin reduced the filtration fraction without increasing the renal vascular tone; however, urinary adenosine and prostaglandin concentrations were increased. The increased adenosine concentrations might be associated with the renin–angiotensin system (RAS) blockade-induced post-glomerular vasodilatation; therefore, renal vascular resistance was not altered [29], which strongly related to post-glomerular vasodilation and not preglomerular vasoconstriction [28].

T2DM hyperfiltration is more complex than T1DM because of additional interactions by metabolic factors such as insulin resistance and RAS activity. Taken together, the evidence indicates that dapagliflozin reduces the single-nephron GFR while maintains efferent arteriolar dilatation by releasing adenosine. The ameliorating effect of RAS activation by adenosine might play a role in treating T2DM hyperfiltration. However, further clinical relevance related to the alterations of SGLT2i should be investigated [5,30].

### 3.2. Effects of SGLT2i on Systemic and Intrarenal RAS

#### 3.2.1. Effects on Systemic RAS

Inadequate RAS activation is crucial in the development of cardiovascular disease and chronic kidney disease (CKD) associated with diabetes [31]. In response to declining renal perfusion pressure and sodium delivery to distal tubules, juxtaglomerular cells markedly increase renin secretion [32]. SGLT2i can cause natriuresis in the early phase of treatment, which may activate systemic RAS. In a nondiabetic CKD rat model, plasma RAS activity was not significantly affected by chronic SGLT2i administration [33].

Clinical symptoms of polyuria and polydipsia develop in the early stages of SGLT2i treatment, indicating enhanced diuresis and obvious negative fluid balance [34]. A study on obese rats indicated that SGLT2i induced short-term loss of body fluids and apparent natriuresis [35]. A clinical study on the long-term use of SGLT2i in patients with T2DM revealed increased urine sodium excretion for only a few days and not thereafter [36]. Thus, the SGLT2i-induced renal diuretic effect can be adequately compensated through an efficacious physiological response.

Patients with T1DM treated with SGLT2i exhibited increased urine angiotensinogen excretion [37], probably due to the activation of intrarenal RAS activity [37]. The authors also found that SGLT2i treatment increased the levels of urine angiotensinogen, ACE, and ACE2 in these patients with T1DM. These findings illustrate that SGLT2i treatment results in extracellular volume depletion, which augments systemic and urinary RAS components, and suggest the activation of intrarenal RAS activated to compensate for an acute volume depletion. However, SGLT2i-treated T2DM rats demonstrated a significant decrease in urine angiotensinogen excretion [38]. Animal studies have also shown that treatment with SGLT2i chronically may not induce the deterioration of kidney function by the activation of RAS in patients with nondiabetic CKD. [33]

A study revealed that SGLT2i treatment did not alter the increased plasma renin activity (PRA) in T2DM mice [39]. Another T2DM fatty rat model indicated that SGLT2 could not suppress the apparent increased circulation of PRA and aldosterone levels [38]. In nondiabetic CKD rats, chronic SGLT2i treatment activated neither intrarenal RAS, as determined by measuring the renal parenchyma angiotensin II concentration, nor systemic RAS, as reflected by the change in plasma RAS parameters such as renin activity and the angiotensinogen concentration [33]. Collectively, these animal studies have shown that SGLT2i treatment acutely increases PRA. However, chronic SGLT2i administration may not influence RAS.

A clinical study in patients with T2DM revealed that SGLT2i treatment for 1 month significantly increased PRA but not plasma aldosterone concentration (PAC). After 6 months, neither PRA nor PAC were changed significantly [40]. These results indicate that SGLT2i did not further activate RAS in the long term, which prevented the RAS-mediated aggravation of cardiovascular and renal events [38,41].

#### 3.2.2. Effects on Intrarenal RAS

The liver is the major source of angiotensinogen in the kidneys [42], and angiotensinogen modulates intrarenal RAS [43]. Furthermore, angiotensinogen is locally synthesized in PTs [44]. SGLT2i acutely but transiently enhances PRA but decreases renal angiotensinogen production through decreased glucose loading in PTs. This can enlarge the glucose load in distal PTs, resulting in augmented angiotensinogen production [41].

PT cells exposed to a high glucose concentration increased angiotensinogen levels [45]. Thus, SGLT2i-mediated decrease in blood glucose levels can reduce angiotensinogen production in the PT, leading to increased glucose delivery to the distal tubule, consequently enhancing angiotensinogen production. A study on Otsuka Long Evans Tokushima Fatty diabetic rats demonstrated intrarenal RAS overactivation in the prediabetic stage, which facilitates the development of diabetic kidney disease [46]. In addition, SGLT2i treatment obviously reduced urinary excretion of angiotensin II and angiotensinogen levels [38].

In patients with T2DM, SGLT2i treatment tended to reduce urinary angiotensinogen excretion [47]. In patients with T1DM, SGLT2i treatment increased urinary angiotensinogen excretion [21]. Several studies have demonstrated a lower urinary angiotensinogen/creatinine ratio in patients with T1DM than in patients with T2DM [48]. This indicated that the baseline intrarenal RAS activity may influence the effect of SGLT2i on intrarenal RAS activity [49].

### 3.3. Ameliorate the Chronic Activation of the Sympathetic Nervous System

Considerable evidence from animal and human studies suggest that overactivation of the sympathetic nervous system (SNS) plays a crucial role in metabolic syndrome [50]. Patients with diabetes, obesity, or hypertension have a high cardiovascular risk, which is related to an inadequate sympathetic nerve activity (SNA) [51]. A meta-analysis revealed that SGLT2i reduces blood pressure [52]. Clinical trials, however, have failed to demonstrate the use of SGLT2i compensatory heart rate (HR) increase [53]. These findings suggest a sympatholytic effect of SGLT2i, which may contribute to the cardioprotective effects of SGLT2i treatment.

#### 3.3.1. SGLT2i Reduces SNS Activity: Animal Data

The SNS plays a crucial role in modulating blood pressure (BP) probably by involving neural signals within the hypothalamus, driven by the central actions of leptin and the activation of brain neurotrophic factors [54]. The SNS is activated in heart failure (HF), an effect potentially deleterious for the heart and kidney [55,56]. An in vivo study found that SGLT2i stabilized the circadian rhythms of blood pressure and SNA and then reduced blood pressure [57]. Thus, nocturnal hypotension, which commonly occurs in patients with DM, could be alleviated. A recent clinical trial involving SGLT2i provided clinical relevance that SGLT2i could normalize BP in the non-dipper pattern, which may provide beneficial effects on cardiovascular outcomes in patients with T2DM [58,59,60].

SGLT2i reduces SNA by different mechanisms. Chiba et al. [61] indicated that SNA originating from the brown adipose tissue could be decreased by SGLT2i. Dapagliflozin treatment suppressed norepinephrine turnover in the brown adipose tissue. Yoshikawa et al. [62] revealed that SGLT2i improved baroreflex sensitivity by lowering SNA in DM rats, thus restoring arterial pressure stability. Matthews et al. [63] reported that dapagliflozin reduced the production of noradrenaline in both the kidney and heart of high-fat-diet-fed mice by reducing inflammatory cytokines such as tumor necrosis factor (TNF)-alpha and interleukin-1B. Jordan et al. [64] found no obvious changes in muscle SNA even with increased natriuresis after short-term treatment of SGLT2i in T2DM. Kusaka et al. [65] demonstrated that SGLT2i did not elicit significant changes in BP in spontaneously hypertensive rats. These results indicate that the inhibition of SGLT2 improves the circadian rhythm of SNA through its sympathoinhibitory effect [49].

#### 3.3.2. SGLT2i Reduces SNS Activity: Human Data

In a human study, SGLT2i mediated calorie loss and diuresis by sodium and glucose reabsorption blockade [66]. Diuretic actions usually promote muscle SNA when the baroreflex is activated [67], and enhanced sympathetic activity is associated with poor cardiac outcomes [68]. However, caloric loss and weight loss are usually linked to decreased muscle SNA [69]. Because SGLT2i could lower caloric and body weight, lowering muscle SNA can contribute to lowering excessive SNA.

A recent study suggested a novel mechanism of SGLT2i-mediated human autonomic cardiovascular regulation [64]: unlike traditional diuretics, SGLT2i did not directly activate SNA. SNA innervates PTs, and its activity can regulate the expression of transporters such as NHE3 and SGLT2 [70]. SGLT2i appears to have beneficial effects on not only morning and evening NP but also nocturnal BP [58,59]. SGLT2i provided greater reductions in BP in patients with higher body mass index and higher baseline BP [71]. Because SGLT2i provided multiple hemodynamic-modulating benefits in RAS and SNS, SGLT2i could significantly reduce cardiovascular events and progression of renal failure in addition to the glucose-lowering effect [58].

#### 3.3.3. SGLT2i Reduces SNS Activity (SNA): The postulated mechanisms

**Figure 3:**
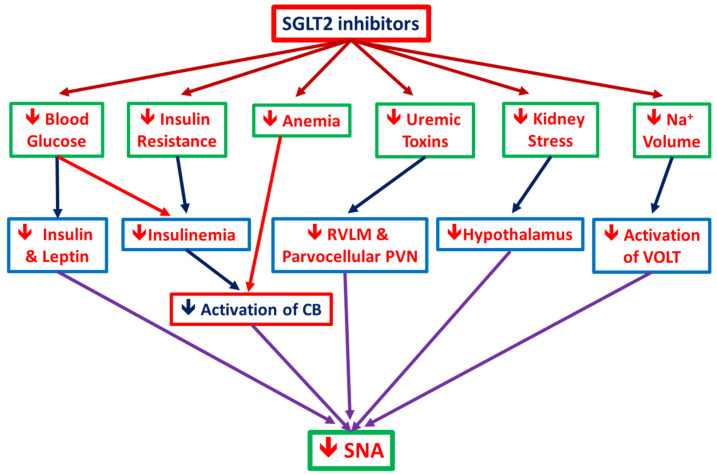
Possible mechanisms of SGLT2 inhibitors on reducing sympathetic nervous activity (SNA): since hypernatremia can activate organum vasculosum laminae terminalis (OVLT) neurons directly, it thus also augments muscle sympathetic nerve activity (SNA) and reduces renal SNA, thereby elevating blood pressure and probably increasing renal natriuresis. SGLT2 inhibitors elicit a reduction in SNA by decreasing insulin, leptin, and blood glucose levels; by improving insulin resistance and hyperinsulinemia; by improving anemia, which could reduce the activation of carotid body (CB); as well as by reducing sodium volume and protein bound uremic toxins level, which inhibits the activation of OVLT in the anteroventral third ventricle (AV3V) region of the hypothalamus.

Figure 3 demonstrated the possible mechanism for SGLT2i in alleviating SNA. In vitro and in vivo studies showed an important crosstalk between the SNA and SGLT2 regulation that might potentially contribute to cardiovascular and renal protection observed with SGLT2 inhibitors [63]. Sympathetic nerves innervate the proximal tubules of the kidney, where they have been shown to regulate the expression of transporters such as NHE3 and SGLT2 [72]. Accumulating data indicated that SGLT2 inhibition might lead to a reduction in SNA, norepinephrine turnover in brown adipose tissue, and tyrosine hydroxylase (rate-limiting enzyme of catecholamine biosynthesis) production. These sympathoinhibitory effects appear to be observed in both animal models of diabetes as well as those with obesity [64,73]. It also has been postulated that the effects of SGLT2i inhibition of SNA might be secondary to a reduction in renal stress with inhibition of renal afferent sympathetic activation [74]. On the other hand, in the central nervous system, SGLT2i may act on the central nuclei-regulated cardiovascular system including paraventricular nucleus of hypothalamus (PVN), nucleus of solitary tract (NTS), periaqueductal gray (PAG), and other nuclei. SGLT-2i may eventually act on rostral ventrolateral medulla (RVLM) to influence the sympathetic flow to the intermediolateral nucleus of spinal cord (IML) with sympathetic preganglionic neurons. Finally, it promotes parasympathetic nervous activity, thereby decreasing blood pressure and heart rate [75].

## 4. Metabolic Factors Influenced by SGLT2i

### 4.1. Pancreatic α Cell Secretion of Glucagon (Hypoglycemia Prevention)

Clinical trials on SGLT2i have revealed that hepatic endogenous glucose production increases as the plasma glucagon concentration increases [76,77]. Both SGLT2 and -1 isoforms exist in pancreatic alpha islet cells [78]; therefore, the postulated effects of SGLT2i on pancreatic islet cells involve the direct inhibition of SGLT2 or compensatory glucagon release during glucose decline. However, results have been inconsistent because SGLT2 expression differs within different models. In an animal model, SGLT1 was the major isoform within the α islet cells [79] and it induced glucagon secretion through α islet cells during treatment with dapagliflozin. Solini et al. also demonstrated that αTC-1 cells with low SGLT2 expression increased glucagon secretion after treatment with dapagliflozin [80]. Taken together, the findings indicate that SGLT2i modulates islet cell glucagon secretion, but the interaction between SGLT1 and the downstream kinase requires further investigation.

### 4.2. Uric Acid-Lowering Effect of SGLT2i

DM is often accompanied by metabolic syndrome, which is typically characterized by hyperglycemia, hypertension, hypertriglyceridemia, and increased uric acid [81]. Hyperuricemia results from uric acid overproduction in the liver and soft tissue because of insulin resistance. Fructose is converted to fructose-1-phosphate, and its accumulation eventually causes increased uric acid formation. Adipokine released from the adipose tissue also increases renal uric acid reabsorption [82]. Uric acid is mostly reabsorbed from rate transporter 1 (URAT-1) within PTs [83]. In CKD, impaired glomerular filtration leads to renal excretion of uric acid. At the same time, the chronic use of furosemide enhances uric acid reabsorption in PTs.

Hyperuricemia increases cardiovascular and overall mortality in patients with DM. Lowering uric acid by the xanthine oxidase inhibitor improved overall mortality. According to Lee et al. [84], URAT-1 expression increased along with SGLT2 in fructose-treated mice. In hyperglycemia, GLUT-9 expression at the basolateral side in PTs increased. GLUT-9 is the main uric acid transporter in PT cells in conjunction with URAT-1 [85,86]. Although the direct association between SGLT2 and URAT or GLUT-9 expression is unclear, a clinical trial demonstrated that dapagliflozin lowered the plasma uric acid concentration in patients treated with bumetanide [87]. Further research is warranted to elucidate the relationship between SGLT2 and URAT.

### 4.3. Effects of SGLT2i on Insulin Sensitivity and β-Cell Function

Recent clinical trials have demonstrated the effect of SGLT2i in improving insulin sensitivity. Kutoh et al. found that SGLT2i administration in patients with T2DM lowered body mass index and HOMA-R [88]. SGLT2i and other DM medications, such as the GLP-1 agonist, were also used in patients with hereditary disease, such as Prader–Willi syndrome [89]. Tahara et al. demonstrated that SGLT2i was effective in treating nonalcoholic fatty liver disease in a high fat diet-induced diabetic animal model [90]. In high fat and cholesterol diet-fed T2DM mice, SGLT2i both alone and with metformin improved fatty liver by alleviating hepatic inflammation and oxidative stress. The most postulated mechanism of SGLT2i in improving insulin sensitivity focused on lowering insulin secretion from beta islet cells when the body’s glucose burden decreased [91]. Hepatic de novo synthesis of fatty acid and cholesterol decreased with a decrease in insulin levels. Energy expenditure increases by browning and thermogenesis in the white and brown adipose tissue, respectively [92], which then reduces the M1 macrophage count and downstream interleukin-6 levels [93]. Inflammation within the adipocytes would be alleviated. Moreover, glucagon release from alpha cells is increased, as previously mentioned, and the beta-oxidation of fatty acid increases the plasma keto acid concentration. As mentioned, insulin sensitivity improved when treating DM with SGLT2i [94]. In an in vitro study, Nakamura et al. reported that insulin stimulated protein expression within PT cells by generating ROS [94] and activated cellular ketogenesis. The generated ketone could also relieve the insulin resistance.

### 4.4. Effects of SGLT2i on Serum Electrolytes and Renal Epithelial Transporter Activity

In the DM animal model, increased SGLT2 expression was accompanied with the modified expression of other specific channels. In an in vivo study, Klein et al. found that streptozocin-induced DM rats had hypokalemia, hyponatremia, hypochloridemia, and decreased urine osmolarity compared with control rats [95]. The tubular protein expression of AQP-2, NKCC-2, and UT-A1 increased in DM rats. After treatment with dapagliflozin for 7 or 14 days, urinary osmolarity increased along with decreased urine volume compared with control rats. Following dapagliflozin administration, the expression of UT-A1 increased but that of AQP2 and NKCC2 remained unchanged. The study provided evidence that the expression of proteins governing the medullary concentration activity increased under the pathologic glycosuric state. At the same time, NKCC-2 exhibited compensatory upregulation as the increased urinary flow increased the amount of sodium. Another in vivo study demonstrated that the urine volume decreased along with urine osmolarity in empagliflozin-treated diabetic rats, although the difference in urine osmolarity was not statistically significant. AQP-2 expression also decreased with the increased expression of vasopressin-2 receptor [17]. Masuda et al. treated 8-week GK rats with ipragliflozin and found them to have a higher expression of the vasopressin-2 receptor with no change in the protein expression of AQP-2 [96]. Taken together, the results indicate that the urinary concentration ability was impaired in diabetic rats. SGLT2i treatment partially improved the urinary concentration ability through osmotic diuresis, even though AQP-2 activity was abated. The vasopressin-2 receptor activity was activated when diabetic rats were treated with SGLT2i. Additional studies are required to understand if SGLT2i modifies vasopressin-2 receptor and its downstream activities.

### 4.5. Effects of SGLT2 Inhibition on Kidney Pathological Findings

As mentioned, the SGLT2 expression within PTs increased with the increase in glomerular hyperfiltration, which induced mesangial expansion. The ECM increased with the deposition of fibronectin and type IV collagen, further damaging podocytes and worsening glomerulosclerosis. High sugar infiltration through SGLT2 could activate the apoptotic-associated protein within PT cells [97]. Vallon et al. reported that glomerular fibrosis or injury was not alleviated in SGLT2-knockout diabetic mice [4]. SGLT2i could modulate oxidative stress and intraglomerular inflammation and could thus alleviate renal fibrosis [98]. It has been well known that the acute kidney injury mediated by drug involves generation of the redox oxidative species, which disturbed the oxidative phosphorylation and mitochondrial membrane. In the glutathione-depleted status, the intracellular ROS accumulation might activate mitogen-activated protein kinase and p53 [99]. The chronic exposure of oxidative stress activated downstream inflammation [100] and further tubulointerstitial fibrosis-related genes such as TGF-β1 and renin-angiotensin-aldosterone system related genes [101]. The vanin-1 expression, which increased after ROS accumulation, has served as the biomarker for oxidative stress within the kidney [99]. From the study by Oraby et al., SGLT2i alleviated the generation of vanin-1 [102]. SGLT2i also lessened the epithelial-to-mesenchymal transition by modulating miR21 [103]. Levi et al. found that lipid metabolism within renal tubules was altered in diabetic nephropathy [104]. SGLT2 inhibition alleviated renal fibrosis by lowering lipid accumulation-induced inflammation mediated by CD68 macrophages [105]. Thus, the podocyte injury could be reversed by modulating Wilms′ tumor 1 gene. Aperia et al. found that high glucose directly induced mesangial and podocyte apoptosis but that SGLT2i could protect against apoptosis even when the expression of SGLT2 within mesangial cells and podocytes was low [97]. SGLT2i also alleviates apoptosis by increasing autophagosomal formation within glomerular mesangial cells and podocytes. Korbut et al. revealed that, in db/db mice, SGLT2i alone or with a DPP-4 inhibitor restored the glomerular autophagosomal formation [106]. Han et al. also demonstrated that SGLT2i, along with thiazolidinedione, could alleviate the glomerular tuft area and mesangial expansion by reducing angiotensinogen and type 1 cytokine [107].

### 4.6. SGLT2i Contributes to Cardiac and Renal Metabolism

In PTs, the resorbed sodium and glucose and thus SGLT2 inhibition lower NHE3 activity [70]. The abated sodium–glucose cotransporter activity leads to the upregulation of other sodium-mediated cotransporters, including the sodium–phosphate transporter or the URAT transporter.

In cardiomyocytes, NHE1 is the major exchanger isoform modulating sodium–proton exchange. An vitro study by Uthman et al. revealed that SGLT2i modulated myocardial fibrosis by inhibiting NHE1 activity, which reduced calcium influx into the myocardium and, consequently, mitochondrial damage [108].

However, SGLT2 might modulate nutrient availability in cardiomyocytes and might influence the cardioprotective effect. During stress, such as starvation or hypoxia, sirtuin-1 (SIRT1), a redox-sensitive nicotinamide adenine dinucleotide-dependent enzyme, is activated to maintain the glucose level [109,110]. The SIRT-1-mediated effect is associated with its interaction with HIF-1 and HIF-2 [111]. In renal tubular cells, SIRT-1 interacts with HIF-2α within the liver and kidney and enhances Erythropoietin (EPO) production. SGLT2 inhibition directly activates gluconeogenesis and ketogenesis through conjunction with SIRT-1 [112,113]. Unlike the dominance of HIF-2α in the renal tissue, HIF-1α is the major isoform that governs the inflammatory process in cardiomyocytes [114,115]. SGLT2 inhibition decreases HIF-1-associated inflammation. During cellular starvation, SGLT2 inhibition also activates autophagy in the myocardium [116,117]. Taken together, the evidence indicates that SGLT2i exerts a cardioprotective effect by regulating energy metabolism and by activating autophagy when cells are in the starvation state following a decrease in the body glucose burden. Among the various metabolic pathways influenced, SIRT-1 is a vital mediator.

### 4.7. Anti-Inflammatory Effects of SGLT2i

In diabetes, chronic inflammation is common because of insulin resistance and uncontrolled adipokine release. Clinical trials have shown that SGLT2i treatment can lower the levels of several cytokines such as tumor necrosis factorα (TNFα), interleukin-6, high-sensitivity C-reactive protein, and leptin [118,119]. The immunomodulation mechanisms of SGLT2i are multifactorial, which are mostly mediated by improving glucose control and body weight decrease.

SGLT2 inhibition can alleviate cardiac inflammation by modulating the phenotype of macrophages. In post-myocardial infarcted Wistar rats treated with dapagliflozin, proinflammatory macrophage and downstream cytokine could be lowered [120]. SGLT2i also reduced cardiac oxidative stress by reducing advanced glycosylated end products within the myocardium or aorta [121,122]. SGLT2i also counters insulin resistance by increasing insulin clearance, thereby abating the inflammatory process [123,124]. Reduced inflammation can also be achieved by SGLT2i’s uric acid-lowering effect [125]. Because SGLT2 is not expressed in immune cells, immunomodulation might be achieved indirectly by controlling DM. Therefore, use of SGLT2i is crucial in controlling chronic fibrotic events involving inflammation.

### 4.8. SGLT2i Reduces Renal Fibrosis and Enhances EPO Production

Erythropoietin is released from peritubular interstitial fibroblasts. In normoxemia, the hypoxia-induced factor is degraded by ubiquitin and inhibits EPO production; also, HIF-2α activity is inhibited by being labeled with the von Hippel–Lindau (VHL)-E3-ubiquitin ligase complex and nordoxepin iron-dependent HIF prolyl-4-hydroxylases. The hypoxemic state produces ROS, promotes HIF-2α translocation into the nucleus, and enhances EPO production by modulating the kidney-inducible element [126]. In hyperglycemia, the oxygen-sensing activity around peritubular cells is disturbed. The excessive glucose reabsorption mediated by SGLT2 overexpression reduced partial oxygen pressure within the peritubular microenvironment. O’Neil et al. demonstrated that SGLT2i administration restored oxygen partial pressure within the cortex, but the medullary oxygen partial pressure remained unchanged [127]. SGLT2i might restore oxygen supply, thereby alleviating the metabolic stress state in the mitochondria and restoring the hematocrit level in patients with DM [128]. Furthermore, SGLT2i reduced ECM fibrosis by inflammation reduction and RAAS overactivation [129]. The EPO-producing peritubular fibroblasts might be preserved. Taken together, the evidence indicates that the EPO-producing ability in patients with DM might be reversed after treatment with SGLT2i [66]. The increased hematocrit may provide an additional cardioprotective effect by augmenting oxygen delivery in patients with congestive heart failure.

### 4.9. SGLT2i in Acute Kidney Injury

SGLT2i attenuate renal fibrosis in diabetic nephropathy by reducing RAAS activation, SNA, and glomerular hyperfiltration. The role of SGLT2i and its interactions in acute kidney injury remain unclear. Recently, Chang et al. noticed that SGLT2i alleviated renal damage in the ischemia–reperfusion animal model through reduction of renal tubular cell apoptosis by increasing HIF-1 and associated proteins [130]. Nishiyama et al. stated that SGLT2i treatment attenuated renal fibrosis in the ischemia-reperfusion rat model by modulating the expression of vascular endothelial growth factor (VEGF). SGLT2i treatment restored the expression of VEGF-A, which ameliorates the endothelial rarefaction of peritubular capillary beds. Use of SGLT2i reduced serum creatinine levels and increased creatinine clearance [131]. However, the clinical relevance of SGLT2i in acute kidney injury (AKI) prevention remains unclear and warrants epidemiological studies on SGLT-2i use and AKI incidence. Moreover, the protective effect from in vitro and in vivo studies should be verified.

### 4.10. Effects of SGLT2i on Bone Metabolism

Bone health relies on calcium and phosphate homeostasis, and the bone remodeling process is mediated by osteoblasts and osteoclasts. In patients with DM, metabolic syndrome and insulin resistance reduce osteoblast-mediated bone resorption, which causes ancient osteocyte clearance. The advanced glycosylated end products deposit within the collagen and impair bone quality, thus increasing the risk of fracture in patients with DM even with the same bone mineral density [132,133]. In diabetic nephropathy with proteinuria, albuminuria itself enhances renal loss by reducing vitamin D reabsorption from PTs by megalin. Moreover, the decreased renal production of 1.25(OH)2D by 1-alpha hydroxylase lowers the endogenous production of active vitamin D [134]. Clinical trials have shown that dapagliflozin does not increase the risk of fracture in patients with DM compared with placebo. At the same time, the bone remodeling marker was not influenced [135]. However, in the CANVAS clinical trials, patients receiving canagliflozin had a higher risk of fracture than the placebo group (hazard ratio: 1.26; 95% confidence interval: 1.04–1.52) [136], which might be related to an increase in abnormal type 1 collagen and body weight loss. SGLT2i was found to increase serum phosphate along with elevated intact parathyroid hormone and decreased 1,25(OH)2D in patients with estimated GFR > 45 mL/min [137]. SGLT2i may enhance proximal sodium–phosphate reabsorption with an increase in the FGF-23 released from osteocytes and intact parathyroid hormone. The body’s phosphate burden is increased [138]. The exact mechanisms underlying SGLT2 and bone health requires further investigation.

### 4.11. SGLT2i on Non-Osmotic Sodium Storage and Interstitial Fluid Dynamics

The natriuretic effect of SGLT2 could lower the sodium burden in patients with DM. Notably, patients with DM have a higher incidence of asymptomatic heart failure because of excessive sodium intake and decreased sodium excretion as diabetic nephropathy progresses. Sodium distribution in the body can be divided mainly into osmotically active sodium and non-osmotic sodium [139]. In salt-sensitive hypertension, the accumulation of Na^+^ in tissue has been presumed to be accompanied by a commensurate retention of water to maintain the isotonicity of body fluids. Titze et al. suggested that subcutaneous local hypertonicity is sensed by macrophages, which then produce the angiogenic protein vascular endothelial growth factor-C (VEGF-C). VEGF-C stimulates lymphatic vessel growth, creating a third fluid compartment that buffers the increased body Na^+^ and volume and ameliorates the high blood pressure. These studies indicate that uniquely stored sodium, the skin subcutaneous lymphatic vessels, and macrophages contribute to volume homeostasis and blood pressure control [140,141]. Thus, Sunitinib, an antiangiogenic, anticancer agent, blocks vascular endothelial growth factor receptors and resulted in increases blood pressure [142]. It has been reported that the skin sodium content was intricately linked to left ventricular mass in patients with CKD. Interventions that reduce skin sodium content might improve cardiovascular outcomes [143]. Furthermore, the sodium retained within the soft tissue increased in patients with chronic renal failure and T2DM who are vulnerable for systemic sodium retention [144]. A recent randomized controlled trial of 6 weeks of dapagliflozin therapy in T2DM patients showed a significant decrease in tissue sodium content (measured by ^23^Na magnetic resonance imaging). This observation points to a decrease in total sodium content among patients with type 2 diabetes prone to cardiovascular complications, that might be mitigated by SGLT-2 inhibition [145]. In patients who received either the SGLT2i dapagliflozin or loop diuretic bumetanide, using the mathematical model illustrated that dapagliflozin had a greater reduction in interstitial fluid (IF) volume compared to blood volume. Dapagliflozin produces a 2-fold greater reduction in IF volume compared to blood volume, while the reduction in IF volume with bumetanide is only 78% of the reduction in blood volume. Thus, by reducing IF volume to a greater extent than blood volume, SGLT2i inhibitors might provide better control of congestion without reducing arterial filling and perfusion [146].

### 4.12. Uremic Toxin-Lowering Effect of Relative Nonspecific SGLT2i

CKD patients with DM will have increased risk of progression to end-stage renal disease, cardiovascular morbidity, and all-cause mortality [147]. Uremic toxins, such as indoxyl sulfate (IS) and p-cresol, or p-cresyl sulfate (PCS), have markedly accumulated in the organs of CKD patients. These toxins can induce inflammatory reactions and can enhance oxidative stress and insulin resistance, prompting the decline of renal function and cardiovascular functions. These deleterious effects could be mitigated at least in part by AST-120 [148]. A recent study from CKD mice demonstrated that canagliflozin, a SGLT2i with a modest inhibitory effect on SGLT1, can influence the gastrointestinal milieu. A two-week treatment significantly reduced the plasma levels of IS and PCS in CKD mice. In addition, its promotion of bacterial carbohydrate fermentation resulted in significantly increased cecal short-chain fatty acids. 16S rRNA gene sequencing of the cecal contents disclosed the increased abundance of the actinobacteria and TM7 phyla, which could be recovered in this SGLT2i treatment group. The altered microbiota composition contributed to the lowering effects on plasma IS and PCS levels [149]. Another recent experiment in CKD rats showed the clue that canagliflozin could ameliorate adenine-induced CKD, through attenuate inflammatory and oxidative stress and lowering plasma IS and PCS, and declined the increase renal content of nuclear factor erythroid 2-related factor 2 (Nrf2). Kidney histology in this SGLT2i treated rats showed less dilated tubules, interstitial inflammation, and atrophic tubules apoptotic cells accompanied by significant improvement in the interstitial fibrosis. Thus, the reduction of accumulated uremic toxins by this kind of SGLT2i could provide a potential therapeutic option in CKD.

## 5. Effects of SGLT2i on Clinical Parameters and Outcomes in Patients with T2DM

### 5.1. The Protection of SGLT2i in Cardiac and Kidney Outcomes

**Figure 4:**
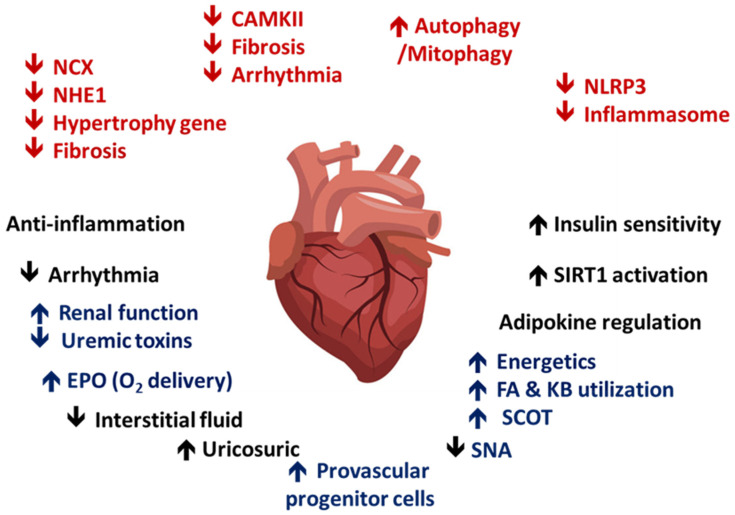
Direct (red) myocardial and indirect/systemic (blue) effects of SGLT2i: the cardioprotective effect of SGLT2i. SGLT2i could alleviate cardiac fibrosis by modulating autophagy and inflammation. SGLT2i also indirectly alleviates cardiac injury by modulating metabolism and the sympathetic tone.

On the basis of the aforementioned molecular mechanisms, SGLT2i provides numerous clinical benefits when administered to patients with DM. They provide considerable benefits in heart failure-associated adverse events. The DAPA-HF study focused on the effect of dapagliflozin in treating DM with reduced ejection fraction. HF-induced cardiovascular mortality and hospitalization decreased in patients receiving SGLT2i [150]. The DECLARE-TIMI58 study demonstrated the benefits of dapagliflozin on major adverse cardiovascular events in patients with DM. In the dapagliflozin-treated group, the incidence of major adverse cardiovascular events remained unchanged but cardiovascular death was lower [151]. The beneficial effects on cardiovascular events have also been demonstrated for other SGLT2i. The CREDENCE study demonstrated that patients with DM who received canagliflozin had decreased cardiovascular mortality and a lower incidence of end stage renal disease (ESRD) or entry of renal replacement therapy during the 2.62-year follow-up duration [152]. The DECLARE-TIMI study also demonstrated that renal outcomes, such as GFR loss or entry into dialysis, were lower in the dapagliflozin-treated group. Recently, the protective effect of SGLT2i in nondiabetic CKD has been evaluated. The EMPEROR-Reduced trial showed that SGLT2i provided renal protection in congestive heart failure patients without DM [153]. In addition to improvements in primary outcomes, such as hospitalization due to heart failure or cardiovascular death, the annual rate of decline in the renal function was slower in the empagliflozin group than in the placebo group among heart failure patients with functional class higher than II (−0.55 mL/min/1.73 m^2^ of the body surface area in empagliflozin group vs. −2.28 mL/min/1.73 m^2^ of the body surface area in the placebo group, *p* < 0.001). The DAPA-CKD trial [154,155] revealed crucial findings in the role of SGLT2i in patients with CKD, especially in patients without DM. Among 4304 patients (67.5% with DM), the occurrence of cardiovascular mortality, decline of glomerular filtration > 50%, and occurrence of ESRD were lower in the dapagliflozin group. The hazard ratio for the primary endpoint was 0.61 (95% CI, 0.51–0.72; *p* = 0.000000028). Thus, the renoprotective effect of SGLT2i, even in patients without DM, was demonstrated in these two landmark studies. Considering the pleiotropic effects of SGLT2i, its application can be broadened (Figure 4).

### 5.2. The Concerns for SGLT2i-Induced Ketoacidosis: Volume Depletion and Insulinopenia

It is likely that plasma volume contraction due to natriuresis in response to SGLT2 inhibition is, at least in part, responsible for the reduction in the risk of heart failure. Other diuretic classes, including thiazide and loop diuretics, have not resulted in such robust clinical benefits among patients with type 2 diabetes, possibly because these agents do not influence intraglomerular pressure directly and obviously. In contrast, SGLT2i inhibitors do have important physiological similarities with carbonic anhydrase inhibitors, which also act proximally, and have been shown to activate tubuloglomerular feedback [156]. Previous study showed a significant increase in urinary pH in Sprague Dawley (SD) rats after luseogliflozin administration [157]. Further immunofluorescence experiments showed that NHE3 colocalizes with SGLT2 in the rat renal proximal tubule. Pharmacologic inhibition of SGLT activity by phlorizin produced a marked inhibition of NHE3 resulting in accentuation of in bicarbonaturia. Therefore, SGLT2i not only downregulates SGLT2 but also decreases sodium uptake by NHE3, thus lowering the reabsorption of sodium and glucose, with consequent improvement of fluid retention, glycemia, and increased urinary pH. However, this systemic acid–base effect is trivial, since it is associated with compensatory increase in renal mRNA expression of genes involved in proximal tubule ammonium, glucose, and bicarbonate synthesis as well as distal tubule H^+^ and ammonia secretion.

Diuretics is commonly used in the diabetic patients with fluid retention. Because of the diuretic effect, doses of other diuretic medications should be revised and, in many cases, decreased in dose when starting SGLT2i in DM patients with CKD. In elderly or frail patients with possible lower water intake or access, SGLT2i therapy should be carefully initiated and closely monitored [158]. From the pharmacological viewpoint, SGLT2i can be regarded as proximal tubular diuretics. If it was used concomitantly with loop diuretics (furosemide), distal tubular diuretics (thiazide), or collecting tubular diuretics (amiloride, triamterene, spironolactone etc.), it might accentuate the diuretic effects acutely in the first few days and aggravate the volume insufficiency. Conversely, as the diuretic effect of SGLT2i is modest, they might add trivial diuretic effects to loop diuretics. Analyses the benefits of dapagliflozin in DAPA-HF study revealed that such a diuretic effect was irrespective of use of background diuretic therapy or dose of diuretic therapy [159]. In sum, the clinicians should pay attention in the DM patients with concomitantly use of diuretics and SGLT2i.

A multicenter cohort study in patients with type 2 diabetes showed that SGLT-2 inhibitors were associated with an almost 3-fold increased risk for diabetic ketoacidosis (DKA), and the molecular-specific analyses suggest it as a classic effect. [160,161]. The pathogenesis of SGLT2i-associated ketonemia is distinct from traditional DKA. SGLT2i promotes elimination of glucose and leads to a decrease in fasting and postprandial serum glucose concentration, which in turn reduces insulin secretion [162]. Glucagon secretion is enhanced from pancreatic α-cells, as the previous section mentioned [79]. Hyperglucagonemia and hypoinsulinemia lead to free fatty oxidation in the liver and resultant ketonemia [163]. In addition, SGLT2i is believed to decrease ketone elimination by the kidneys [162]. In adults with T1DM, adding SGLT2i to insulin reduces hemoglobin A1c levels and body weight but increases diabetic ketoacidosis and genital infections [164]. Recent studies proved that the SGLT2i dapagliflozin promotes ketoacidosis in both healthy and type 2 diabetic rats in the setting of insulinopenia through increased plasma catecholamine and corticosterone concentrations secondary to volume depletion [165]. These derangements altogether increase the white adipose tissue (WAT) lipolysis and hepatic acetyl-CoA content and increase hepatic glucose production and hepatic ketogenesis rates. Treatment with a loop diuretic, furosemide, under insulinopenic conditions replicates the effect of dapagliflozin and causes ketoacidosis. Taken together, these data in rats identify the combination of insulinopenia and dehydration as a potential target to prevent euglycemic ketoacidosis associated with SGLT2i.

## 6. Conclusions

SGLT2i is essential for treating patients with DM because of its pleiotropic effects in modulating glomerular hemodynamic stability and metabolic effects involving glucose control and improving insulin sensitivity. In addition to diabetes control, this class of drugs have cardio- and renoprotective effects in patients with or without DM, as demonstrated by both clinical trials and in vivo studies. Additional studies to understand its benefits on multiple organs are required, and it is essential to broaden its applications in patients with chronic cardiac/renal dysfunction with or without DM.

## Abbreviations

ACE	Angiotensin converting enzyme
AKI	Acute kidney injury
AQP-2	Aquaporin 2
AV3V	Anteroventral third ventricle
BP	Blood pressure
CB	Carotid Body
CKD	Chronic kidney disease
DKA	Diabetic ketoacidosis
DM	Diabetes mellitus
DPP4	Dipeptidyl peptidase-IV
ECM	Extracellular matrix
EPO	Erythropoietin
ESRD	End stage renal disease
GFR	Glomerular filtration rate
GLUT2	Glucose transporter 2
GLUT-9	Glucose transporter 9
HF	Heart failure
HIF	Hypoxia inducible factor
HOMA	Homeostasis model assessment
IML	Intermediolateral nucleus of spinal cord
IS	Indoxyl sulfate
NHE3	Na+/H+ exchanger isoform 3
NKCC-2	Na-K-Cl cotransporter 2
OVLT	Organum vasculosum laminae terminalis
PAC	Plasma aldosterone concentration
PAG	Periaqueductal gray
PCS	P-cresol sulfate
PRA	Plasma renin activity
PT	Proximal tubules
PVN	Paraventricular nucleus of hypothalamus
RAS	Renin-angiotensin system
RCT	Randomized clinical trials
ROS	Reactive oxygen species)
RVLM	Rostral ventrolateral medulla
SGLT-2	Sodium–glucose cotransporter 2
SGLT2i	Sodium–glucose cotransporter 2 inhibitor
SIRT-1	Sirtulin-1
SNA	Sympathetic nerve activity
SNS	Sympathetic nerve system
T1DM	Type 1 diabetes mellitus
T2DM	Type 2 diabetes mellitus
TGF	Tubular-glomerular feedback
TGFβ	Transforming growth factor β
TNFα	Tumor necrosis factor α
VEGF	Vascular endothelial growth factor
VHL-E3	Von Hippel-Lindau ubiquitin ligase-E3
URAT1	Urate transporter 1
